# What do we know about the impact of the Covid-19 pandemic on hospices? A collaborative multi-stakeholder knowledge synthesis

**DOI:** 10.12688/amrcopenres.13023.1

**Published:** 2021-10-06

**Authors:** John MacArtney, Abi Eccles, Joanna Fleming, Catherine Grimley, Jeremy Dale, Kathryn Almack, Catriona Mayland, Sarah Mitchell, Ruth Driscoll, Rebecca Hammond, Lynn Tatnell, Lesley Roberts

**Affiliations:** 1 Unit of Academic Primary Care, University of Warwick, Coventry, Warwickshire, CV4 7AL, UK; 2 Communities, Young People and Family Lives, University of Hertfordshire, Hatfield, Hertfordshire, AL10 9AB, UK; 3 Department of Oncology and Metabolism, University of Sheffield, Sheffield, Yorkshire, S10 2TN, UK; 4 Policy and Public Affairs (England), Marie Curie Cancer Care, London, London, SE1 7TP, UK; 5 Patient and Public Involvement Representative, NA, UK

**Keywords:** Covid-19, pandemic, hospices, palliative care, knowledge synsthesis

## Abstract

**Background:**

Prior to undertaking a study looking at the effects of the COVID-19 pandemic upon lived experiences of hospice services in the West Midlands, we sought to identify the range of issues that hospice service users and providers faced between March 2020 and July 2021, and to provide a report that can be accessed and understood by all interested stakeholders.

**Methods:**

We undertook a collaborative multi-stakeholder approach for scoping the range of potential issues and synthesising knowledge. This involved a review of available literature; a focus group with hospice stakeholders; and a collaborative knowledge exchange panel.

**Results:**

The literature on the effects of the COVID-19 pandemic on hospices remains limited, but it is developing a picture of a service that has had to rapidly adapt the way it provides care and support to its service users, during a period when it faced many fundamental challenges to established ways of providing these services.

**Results:**

The impacts of many of the changes on hospices have not been fully assessed. It is also not known what the effects upon the quality of care and support are for those with life-limiting conditions and those that care for them. We found that the pandemic has presented a new normative and service context in which quality of care and life itself was valued that is, as yet, poorly understood.

## Introduction

People with life-limiting conditions are highly vulnerable to COVID-19. Alongside NHS care, they can expect to be supported by a network of informal carers and civil society organisations, including their local hospices. But following the countrywide lockdown in March 2020, hospices, like healthcare across the country, rapidly changed the way they worked, how they cared for patients, and how they supported families ( Oluyase *et al*., 2020). Palliative and end of life care services have been a vital part of the pandemic emergency response, shifting their service towards caring and supporting people with life-limiting conditions in community settings ( Bowers *et al*., 2020; Etkind *et al*., 2020; Sleeman *et al*., 2021).

Each hospice’s response to the COVID-19 pandemic will have reflected local conditions, but common to all in the UK has been the theme of adapting large portions of care and support to a now dispersed community of service users ( Oluyase *et al*., 2020). However, there is good evidence to show that the effects of the pandemic have not been experienced equally across socio-economic groups ( Marmot *et al*., 2021). The pandemic also brought new ways of valuing and discussing life ( Pickersgill, 2020), with those with pre-existing and life-limiting conditions experiencing challenges to the meanings placed upon both the amount of life (predicted to be) left, as well as the quality of those lives ( Driessen *et al*., 2021; Kirby *et al*., 2020). The changes to healthcare policy throughout the pandemic therefore affected not only who lives and dies, but how people with life-limiting conditions lived during the pandemic, how they died, and how this was experienced by families, carers, and hospice staff.

Our aim for this report is to identify the range of issues that hospice service users and providers have faced between March 2020 and July 2021. As we do so, we identify what else can be done to help and support hospices and their service users as well as highlight any gaps in in the evidence. Some of the issues we address have already been explored by other researchers and their invaluable work and relevant recommendations are noted within this report. However, we have found that there are only a few studies specifically examining the changes to hospice services and their impact on the lived experiences of receiving or providing hospice care during the pandemic. This means that there is a lack of understanding about how, when, and for whom the changes to palliative care have been beneficial. As we explore below, we have found that there remains an urgent need to gather evidence of the on-going impact of the COVID-19 pandemic on hospice care, and use this to inform the current and future design and delivery of hospice services end-of-life care.

### Background to this report

This report is written for all those who have a stake in hospice services, from patients, their families and those that care for them, through to clinical and non-clinical staff and the charities that run many hospices, as well as service commissioners and those that oversee healthcare policy and provision in the UK. It is the first output from an Economic and Social Research Council funded study (grant number: ES/W001837/1) that will contribute the missing hospice perspective to the growing body of knowledge about the effectiveness and effects of changes to hospice services, at regional and national levels in response to COVID-19. This study seeks to provide understanding and recommendations to mitigate the uneven relational, social and healthcare impacts of COVID-19 upon hospice services. In the main phase of the study, we will use two data collection methods: the first involves the collection of already existing quantitative and qualitative data and outputs created by the hospices in response to the pandemic. The second comprises in-depth interviews with patients, carers, hospice staff, and with those responsible for hospice service design and provision.

However, before undertaking a large study like this, researchers would ordinarily conduct a systematic review of the literature to identify key issues or questions they are seeking to address. However, as the main study seeks to provide a rapid response to a novel pandemic, we were not able to conduct a systematic review of the relevant literature or engage the public, patients or stakeholders, prior to developing the main study. Therefore, the primary aim of this pre-study phase was to identify the issues or themes we might anticipate exploring in the remaining months of the main study, as well as any gaps in the existing evidence that we might be able to address. To do this we used the resources available to us at the time, which were: reading the developing literature; engaging stakeholders; and identifying priorities with subject and experiential experts in the hospice field. By sharing what we found in this report, we also hope to be able to provide a snapshot of how the COVID-19 pandemic is affecting hospices in the UK.

## Methods

The methodological design of this pre-study phase of the study involved a collaborative multi-stakeholder approach to scoping the field and synthesising knowledge. This is a form of ‘live’ methods (e.g. Back & Puwar, 2012) that is situated within an emergent and uncertain context, but seeks to provide a near real-time considered evaluation of what has and is happening. Our approach positions the multidisciplinary research team – which includes general practitioners, specialist palliative care (hospice) consultant, health scientists, psychologist, sociologist, policy and patient and public involvement (PPI) representatives – as interested stakeholders in the field of hospice care. By engaging with the multiple disciplines of the team and stakeholder interests of those we collaborate with, we seek to locate the emerging developments within wider systemic social and healthcare perspectives, as well as within pre-pandemic trends in hospice care and research. We therefore scoped the potential issues that hospices and their service users were facing through three data engagement methods. First, a review of available literature; second, a focus group with hospice stakeholders; and, thirdly, a collaborative knowledge exchange panel. This paper conforms to Standards for Reporting Qualitative Research (SRQR) guidelines.

### Literature review on the pandemic and hospice care

We first sought to identify and collate the developing literature on the impact of the Covid-19 pandemic upon hospice care in the UK. We considered it premature to conduct a systematic review of the literature on the pandemic and hospices, given the limited timeframe for researchers to conduct studies and publish findings, and the fast pace at which such literature was appearing. Between April 2021 and July 2021, we purposefully sought to identify all articles related to “hospices” and “Covid-19” via Google Scholar, Twitter and through co-author academic and professional networks. The types of literature we reviewed included peer-reviewed articles, as well as pre-prints, policy documents, and third sector reports. Further to this, we purposefully sought literature that could provide transferable insights from related but wider healthcare fields. This included studies exploring the impact of the pandemic on healthcare staff and service users, and studies looking at the social and healthcare inequalities related to the pandemic.

### Stakeholder engagement focus group

Ethical approval was provided by the lead author’s institution (approval number BSREC 98/20-21) for the whole study, which included stakeholder focus groups and the knowledge exchange panel. An online stakeholder meeting was held with nine participants, including hospice clinicians and research facilitators, invited by email from across the West Midlands hospice research community, who expressed an interest in supporting the main study. We introduced the study and facilitated a discussion about the impact of the pandemic on hospices. Verbal consent was provided by the participants for us to record the event on Microsoft (MS) Teams, but not transcribed, so it could be reviewed to ensure no issues, themes or nuances were missed. The themes and issues identified were summarised in a short report and then cross-referenced with the themes identified in the literature. A short lay summary of these themes was then generated in preparation for the knowledge exchange panel.

### Knowledge exchange panel

The knowledge exchange panel lasted half a day (3.5 hours) and involved 11 participants drawn from the research team, including the PPI representatives. The initial goals of the panel were to identify and prioritise the themes, issues or questions to be explored that will provide the greatest insight into the uneven relational, social and healthcare impacts of COVID-19 upon hospice services for service users and those that care for them. Verbal consent was given for meeting to be recorded on MS Teams and was transcribed using MS Stream software.

To identify and prioritise the issues and questions, we used a modified nominal group technique (M-NGT) ( Manera *et al*., 2019), a collaborative and consensus building approach, to generate transferrable insights, understanding and recommendations. NGT is particularly suited to working with participant groups with diverse experiences of an issue, such as those containing clinicians, patients, and close-person carers, as it is structured to ensure equal participation and prevents dominance of one voice ( Carney *et al*., 1996).

A NGT has four stages, with a M-NGT allowing adaptations to one or more stages ( Manera *et al*., 2019). For this study we circulated a summary of themes identified from the literature review and stakeholder meeting to allow participants to reflect on amendments to the themes identified (stage 1). Having ensured we had everyone's consent, the meeting started with, a round-robin where participants were each asked, in turn, if there were any amendments to the themes or issues identified in the summary report (stage 2). Once all stakeholder amendments were noted, (stage 3) the floor was opened-up to allow other participants to question and discuss the suggested amendments. A diversity of views and interpretations were encouraged, with differences of interpretation included in the findings. In the second half of the discussion the facilitator (JM) led the drawing up of a shortlist of themes, issues or questions affecting the uneven relational, social and healthcare impacts of COVID-19 upon hospice services for service users and those that care for them. The last stage of the M-NGT (stage 4) involved ordering the shortlist to prioritise issues for the Research Team to consider.

### Generating this report

By the end of the knowledge exchange panel, the participants had agreed on a list of themes and issues that we anticipated the main study would focus on. Two of the researchers (AE, JF) then went back through the knowledge exchange panel transcription to extract and paraphrase the panel’s comments to substantiate discussion points, as well as returning to the literature and Stakeholder Event to similarly identify and extract all relevant evidence. JM, AE and JF then collated this evidence into a first long draft of the findings. This was shared with the co-authors, including the PPIs, to confirm interpretations. Using this feedback, JM then developed the first draft of this report and led the redrafting up to submission.

The discussion at the knowledge exchange panel sought to explore themes for each cohort – patients, carers, staff, hospice service providers – as well as identifying any cross-cutting issues. As the discussions progressed two things became evident: first, it was more important to understand how each of the themes was pertinent in the context of hospice care, than it was to prioritise any one issue over the other; second, it was also evident that no theme affected just one cohort, and that the way any issue or challenge affected each cohort was related to and dependent on how it affected the other cohorts.

## Results

### Hospices: an overlooked service

One of the major concerns during the pandemic is the strain it puts on healthcare services, especially when rates of COVID-19 infections within the population are at their peaks. Much of the media attention, and government decisions, focussed on overwhelmed hospitals and intensive care units (ICUs). However, there was less attention afforded to other settings where a substantial amount of health care, including palliative and end of life care, took place – community health and social care services such as primary care, district nursing, or care homes ( Bowers *et al*, 2021; Mitchell *et al*., 2021; Oluyase *et al*., 2020). In particular, non-NHS hospice services experienced rapid and sizeable changes affecting all aspects of care and support ( Sleeman *et al*., 2021).

Participants at the stakeholder event described how they felt the role and importance of palliative care had been excluded by the government and the media. Providing hospice care during the pandemic has been incredibly challenging and this was further compromised by shortages of essential PPE, medicines, and staff. There was a view that this was made worse by hospices not being seen as ‘frontline NHS’ (see also, Sleeman *et al*., 2021). Some participants reported feeling that the extra work done above-and-beyond normal duties had not been adequately recognised. If hospices had come under the remit of NHS services, it was observed, they might have received more attention from local and national government and not been an overlooked service.

As we discuss below, hospices and their service users have experienced many issues in common with community NHS healthcare provision, and care, nursing and residential homes. But hospices have also had to manage particular configurations of these issues. Specifically, as we explore in the second section of the findings, the pandemic presented a multi-layered challenge to the foundational principles of hospice care, including the emphasis upon improving the quality of life for those with life-limiting conditions. Before we turn to these issues, we discuss some of the more distinctive challenges that hospices faced, including the loss of charitable income, the reliance on volunteer workforce, and the issues faced when visiting in-patients in hospice during a pandemic.

***Impact on resources and funding.*** The COVID-19 pandemic has had a substantial impact on resources and funding for hospices. Most hospices are charities and so largely rely on donations, fundraising, and income such as from charity shops. Hospices also use support from volunteers in their day-to-day running. With the closure of charity shops, paused fundraising and loss of volunteers’ support (due to infection risk), hospices have suffered immense strain on income and available workforce. It has been challenging for staff dealing with this lack of resources ( Sleeman *et al*., 2021) and clinical participants at the knowledge exchange panel and the stakeholder event both described concerns of decreasing staff morale in hospices.

The pandemic has also highlighted the non-NHS status of many hospices, as well as the precarity of many hospices funding arrangements with local and national governments. Attendees at the stakeholder event, and a PPI member of the knowledge exchange panel, felt strongly that hospice services ought to be better supported financially by local and national governments, and less dependent on charitable giving. Knowledge exchange panel participants discussed how hospices had to lobby the government to receive some emergency funding, which was put to good use in community palliative support services. But they also expressed concerns about what happened once the emergency funding had ended. The knowledge exchange panel participants reflected on how the pandemic had brought to the fore the need to explore different, more sustainable, funding models for hospice care. While this is a longer-term funding goal, what is also needed was a remodelling of how palliative care might be delivered within the confines of current or near-future funding. In particular, more research is needed on how specialist palliative care in the community can make better connections and alliances with primary palliative care providers.

***Loss of volunteers.*** Many hospices rely on a significant volunteer workforce to deliver their services and support people at the end of life. A rapid review found that during previous pandemics elsewhere in the world the cessation of the volunteer workforce has had significant impact on palliative services, whereas some hospices were able to redeploy volunteers to new roles that provided support to service users ( Etkind *et al*., 2020). During the COVID-19 pandemic, hospices found that there was a tension between the need for volunteers and the infection risk ( Walshe *et al*., 2021). Some hospices did adapt volunteer roles, including remote befriending or bereavement support, driving, delivering, shopping, gardening, as well as using volunteers to support service users with the completion of care plans and communication/coordination of care ( Walshe *et al*., 2021). However, for many hospices finding ways to continue and manage volunteers’ involvement was not a priority, and this led to increased demands on many hospice services and strained their paid workforce ( Walshe *et al*., 2021). These survey findings were supported by several participants of the stakeholder event, who described the loss of volunteer support and how it had caused additional workload strain, as staff members also had to cover volunteers’ duties. Knowledge exchange panel members identified that what remains to be seen is how the volunteers themselves experienced the changes and/or adapted their involvement. Additionally, little is known about the effect on the patients and carers who had come to depend on this voluntary workforce.

***Changes to visiting arrangements.*** In response to this pandemic most health and social care organisations put in place restrictions on who could visit in-patients or residents. At many care homes and hospitals these new visiting rules were found to significantly affect how people were able to say goodbye, from people having final moments separated by a window to not being able to be present at all ( Hanna *et al*., 2021). In an interview study with bereaved relatives, Hanna *et al*. described how visitors faced a conflict between wanting to be with their relative, but also knowing that they should stay away for fear of passing on the virus to the person they were visiting, other in-patients, or staff. Similarly, perceptions about having to wear personal protective equipment (PPE) and practicalities of self-isolation afterwards were identified as barriers to people visiting ( Hanna *et al.*, 2021).

When people were able to visit, the lack of in-person contact that family members experienced brought a number of communication challenges for those involved ( Hanna *et al*., 2021). Close persons’ communication and physical contact were hampered by the PPE (e.g., masks, gowns, screens etc). For many, in-person visits were not possible and so family and carers relied upon regular updates from care home and hospital staff on personal care and condition. While families would want detailed information on prognosis, condition, or symptoms, they reported that they were often just told their family member was “comfortable” ( Hanna *et al*., 2021).

These difficulties experienced in care homes and hospitals were echoed by knowledge exchange panel members and their experiences of hospice-visiting during the pandemic. They reported how at the start of the pandemic visitations in a specialist palliative care unit had been limited to the patient and visitors on either side of a window. However, it was noted that – at that time – this was a less restrictive family-visiting policy than in the local hospital, where this type of visit would not have been possible. Similarly, the stakeholders also shared how challenging it was to witness the impact of restrictions on patients and family members, with some having to choose which two family members could visit at the exclusion of others. They also witnessed the frustrations felt by family members, especially as the pandemic progressed when restrictions had been in place for such a long time. Judging when a patient was in the last 24–48 hours of life has always been characterised by a great deal of uncertainty ( Taylor *et al*., 2017), but stakeholders described how the pandemic situation, use of remote communication, and the restrictions on visitors had made this all the more stressful and distressing for all involved.

The stakeholder reflections help draw attention to the importance of the missing patient experience in the evidence collected so far. This includes both those patients who were in-patients during the pandemic, but also those who may have expected to have had in-patient care but who were unable to access such care. Prior to the pandemic, of those people with life-limiting illnesses admitted to in-patient hospice units for symptom stabilisation and/or pain management, between 5%–23% might return home ( Wu & Volker, 2019). How these palliative care needs were met and patients’ experiences of the quality of this support are therefore in need of further exploration.

### Impact upon the quality of hospice care

Concerns about the detrimental effect on quality of hospice care were found in the literature (e.g. Mitchinson *et al*., 2021), as well as being voiced during the stakeholder event and knowledge exchange panel. It may take several years to properly assess the full effect upon the quality of palliative care. However, it is evident that the pandemic has produced several new challenges, as well as presenting existing issues in new contexts, such as: addressing demographic and geographic inequalities in palliative care; the integration of hospice care and how best to collaborate with other health and social care services in the community; acceleration of hospice at home initiatives; rapid changes and the challenge of identifying what works and what does not; digital and remote ways of providing palliative care and support; and how the COVID-19 pandemic has affected bereavement support, are all important areas for future research.

***Demographies and geographies of care.*** It has been observed throughout the pandemic that COVID-19 has had a disproportionate impact upon particular social, ethnic and economic groups, such as low socio-economic status, ethnic minorities, disabled people and those with pre-existing medical conditions such as multiple sclerosis or HIV ( Marmot *et al*., 2021). Participants at the knowledge exchange panel discussed the potential diversity of communities that hospices serve and suggested that there was a need to ensure more equal access to services for these groups before the pandemic, which the pandemic may have exacerbated. The knowledge exchange panel participants were concerned that the pandemic may have brought some novel issues and magnified other challenges to reaching and supporting people with life-limiting illnesses from these groups. Similarly, participants were concerned that where someone lives might affect the palliative services that are available to them, with those in rural areas potentially more reliant on district nurses than on hospice support.

***Places of care: towards integrated hospice care in the community.*** Hospices have a long-standing association with providing holistic care to patients and families through hospice day centres and in-patient units. While many also deliver care in the community (including in care homes and in people’s homes), the last couple of decades have seen this approach challenged by digital innovation, increased need for generalist palliative care, and community empowering approaches ( Abel, 2018; Clark *et al*., 2020). The pandemic has been a catalyst for these initiatives, bringing immediate changes affecting almost every aspect of providing hospice care and support, from clinical practice to interpersonal and social relations ( Dunleavy *et al*., 2021). The shift in location of care has also led to an expanded case load for community staff, with community nurses having to carry out more roles than usual ( Bowers *et al*., 2021; Mitchell *et al*., 2021). For example, completing do-not-resuscitate forms, being involved in medical decisions, and verifying death certificates ( Bowers *et al*., 2021).

During both the stakeholder engagement and knowledge exchange panel it was observed that the increase in community palliative care had given rise to some overlaps in services between specialist palliative care and primary care. During the knowledge exchange panel, there were strong feelings from participants that the two sectors should work more closely together, and that effective communication would be vital to this. But there was also recognition that such collaboration can be challenging and highly dependent on the specialist palliative care available in a location, and how engaged the local primary care teams are in palliative care. Knowledge exchange panel participants judged that a ‘one -size-fits-all’ approach to such collaboration was inappropriate due to variations in the services available. There were similar concerns from the stakeholders that if integration of care does not work well, there is potential for tensions between specialist palliative care and primary care to arise. Knowledge exchange panel members recognised that increases in funding were unlikely and discussed the need for research into how to transform the way that palliative care is delivered in the community, to enable hospice and primary care to collaborate, which may require a culture change and additional training ( Higginson *et al*., 2021; Mitchell *et al.*, 2021).

***Hospice at home.*** Due to the restrictions on usual forms of hospice care ( Mitchinson *et al*., 2021), there has been an increase in the amount of palliative care being carried out in people’s homes, particularly for care that previously would see patients admitted to hospices or hospitals ( Dunleavy *et al*., 2021; see also, APPG for Terminal Illness, 2021). Significantly, there has also been an increase in the palliative care and support that family members have been expected to provide at home and in care homes ( Sleeman *et al*., 2021). This includes family/carers having to learn how to administer medication and provide care ( Bowers *et al*., 2021; Dunleavy *et al*., 2021). Related to this has been a change of drug administration methods, from subcutaneous at in-patient units to oral at home ( Dunleavy *et al*., 2021; Etkind *et al*., 2020).

Some participants at the stakeholder event said they had been surprised by the extent of the clinical care provided in hospices that could be carried out at home. Participants of the knowledge exchange panel highlighted that while the home is a suitable place for palliative care for some, it is not the best place for everybody. For example, many homes do not have the space for the equipment needed (such as hospital beds). Nor do all those with life-limiting conditions have family and friends who are able or willing to provide the informal and unpaid for labour to support them at home. Further research is needed to better understand how hospices can best address the needs of different parts of the community in their homes.

***Digital and remote palliative healthcare.*** Members of the knowledge exchange panel and stakeholder event, as well as the background literature, all referred to adapting to the use of more remote communication methods (such as phone or video calls) in the absence of in-person contact. This includes remote communication being used for consultation, care planning and patient-family communication, as well as ‘check-ins’ with patients to ensure they have supplies and reassure them that the hospices were still there for them ( Dunleavy *et al*., 2021).

Remote communication also has implications for the quality of palliative care provided and experienced. Hanna *et al*. (2021) reported that family members experienced more negative experiences due to not being able to arrange phone and video calls with the family member or friend who had been admitted to the hospice, as well as finding communicating via phone and video upsetting. At the stakeholder event, we also heard about the difficulties clinicians had in assessing a patient’s condition via a phone call. While some stakeholders noted that they could make better assessments via a video call (as they could see the patient), this was not always a viable option for some patients, either due to digital illiteracy or deprivation. Other stakeholders described feeling frustrated that working remotely has meant that they have not always been able to fulfil their ‘normal’ role of finding solutions to problems faced by patients and carers.

More broadly, the knowledge exchange panel participants discussed the importance of digital community support to help the patient and family. It was noted that there is a lot of information available that is useful for family members (e.g. Marie Curie’s’ support webpages); however information on the Internet is not always easy to find, or provided in ways that everyone or every community might find accessible. It was recognised that there is a need to disseminate this into community services, and to members of the public. Knowledge exchange panel participants highlighted a lack of understanding on the lived experienced of the impact of the move to digital and remote services upon those with life-limiting conditions during a pandemic, especially how these may have been experienced unevenly. For example, the experience of people without access to digital technologies such as laptops or tablet-computers, how older patients adapted to new technologies, or how communication was managed with those whose first language is not English.

***Changes to services that worked, changes that did not work.*** The pandemic and the first lockdown brought rapid changes not only to what services were provided, but also to how they were delivered. The speed of change was noted by some to bring better collaboration and continuity of care, as well as efficiencies in communication between healthcare professionals ( Dunleavy *et al*., 2021). For example, in one area, having a central point of contact for accessing services was instigated and observed to have led to better cross-service collaboration – an initiative that had been discussed in the years before the pandemic and that was quickly and successfully implemented ( Dunleavy *et al*., 2021). This survey described why staff felt some changes were successful, including the need to disregard previous concerns about service changes; how staff and organisations pooled resources across hospices; how they had acted flexibly (due to both a willingness and a need to be flexible); the presence of strong leadership; and an emphasis upon collaborative teamwork, both within and between specialist palliative care services and with other generalist palliative care providers ( Dunleavy *et al*., 2021: 13). It was also noted having a pre-existing IT infrastructure helped, and that some emergency funding had been available to fund the new ways of working ( Dunleavy *et al*., 2021). The changes did not always come easily, and the survey respondents described how they had to overcome several issues, including a lack of IT devices and poor Wi-Fi at some hospice locations and in the community ( Dunleavy *et al*., 2021).

Similarly, participants at the stakeholder event described how several long-discussed initiatives to improve cross-service provision and collaboration between hospices in the region became overnight realities. But not all changes were continued with, nor were all adaptations successful. For example, some stakeholders explained that at the start of the pandemic, they had sought to call all their service users to provide reassurance and continuity of care, as they aimed to find ways to maintain human connections and re-establish compassion towards the end of life ( Etkind *et al*., 2020; Mitchinson *et al*., 2021). But the stakeholders related how, as the weeks progressed, decisions were made to stop these proactive phone calls as they anticipated being overwhelmed. These stakeholders went on to discuss how, in hindsight, they realised this had a detrimental impact as people became reluctant to get in touch with them, as they had assumed the hospices would be busy. The stakeholders felt this meant that they were often not in contact with clients until they were in crisis, and there was a need to carefully balance this desire to find ways to maintain human connections.

The impact of the pandemic on the number of available in-patient hospice beds varied across the country ( Dunleavy *et al*., 2021). In some areas, where need increased, hospices reconfigured space and paused respite beds to increase the number of beds ( Dunleavy *et al*., 2021). Some participants at the stakeholder event described how their hospice decided to stop accepting people from hospitals to prevent importing COVID-19 infections. The knowledge exchange panel were sympathetic to these accounts, noting that if infections were introduced to the inpatient hospice setting and they had to close down, then this would be a crisis for many other patients, who needed specialist in-patient hospice care and could not be cared for as well elsewhere. This had several implications including a loss of income from those who would ordinarily be referred from hospitals, as well as the need to rapidly reorientate their services to provide care in the community settings.

Further, and as already discussed, the use of digital technology gave rise to the need to adapt and experiment with how services could be provided. Dunleavy *et al*. (2021) documented the range of activities carried out via digital communication technology including: YouTube complementary therapy sessions; ‘Time to Create’; volunteer/befriending; telemedicine, electronic care plans; symptom assessments; ward rounds and administration assessments; and virtual visiting. The increase in digital technology use was accompanied by specific challenges, from quickly training staff to use digital technologies to ensuring necessary data protection schemes were in place ( Dunleavy *et al*., 2021: 2).

Finally, advanced or anticipatory care planning (ACP) has been significantly impacted by the pandemic. ACP would usually take place over a series of in-person conversations between clinicians and patients. An evidence review by Selman *et al*. (2020) described how the pandemic brought several barriers to conducting effective ACP in the community. Members of the knowledge exchange panel echoed the review’s concerns, particularly around the difficulty to know what options could be offered in the ever-changing pandemic context. The review recommended that video and web-based ACP be trialled, and sought increased funding for resources to reduce inequalities ( Selman *et al*., 2020).

It is therefore clear that the pandemic brought significant challenges to hospice services and their staff. But as the stakeholders described, there were also significant personal challenges to be faced when delivering changes, from a personal lack of confidence to quickly learning new ways of working. What is missing are the voices of those receiving these new or revised services – the patients and carers. What will be judged a successful ‘improvisation’ ( Dunleavy *et al*., 2021: 2) will depend upon listening to their experiences of affected care and support.

***Impact on bereavement support.*** The pandemic has profoundly affected how people grieve. A recent survey reported high levels of emotional support needs amongst adults bereaved during the pandemic. The majority of survey respondents had not accessed bereavement support services and 39% had difficulties gaining support from friends and family, leading to increased feelings of isolation ( Harrop *et al*., 2020).

With more people experiencing grief, it has been surmised that those with prolonged and complicated grief responses are likely to become more numerous ( Sleeman *et al*., 2021). While some have anticipated an escalating level of need and argued for increased resources to help prevent a ‘tsunami of grief’ ( Pearce *et al*., 2021), others have urged caution against the over-pathologisation of grief and bereavement in favour of a response that is alive to the disproportionate effects of the pandemic on certain groups ( Rose *et al*., 2020). What the pandemic has highlighted is the need for increased attention to bereavement and investment in mental health services, along with better integration of mental health care into palliative care provision ( Pearce *et al*., 2021; Rose *et al*., 2020).

There has been an increase in bereavement support that has been carried out remotely ( Pearce *et al*., 2021). Knowledge exchange participants discussed how normal support mechanisms (such as face-to-face contact with family) were not always feasible. A systematic review of the literature has also shown that those who had difficulties accessing support from close family and friends following other types of mass bereavement events also struggled to cope, but that there were no high-quality studies on the immediate and longer term effects of mass bereavement from a pandemic ( Harrop *et al*., 2020). Nonetheless, there was sufficient evidence to suggest that there ought to be increased investment in bereavement support initiatives to raise awareness regarding the services that are available and how to access them.

## Discussion

By using three data collection methods – the literature review, the stakeholder event, and the knowledge exchange panel – we were able to bring together a range of evidence and perspectives on the impact of the COVID-19 pandemic upon hospices in the UK. Although the literature on COVID-19 and hospices remains limited, it is developing a picture of a service that has had to rapidly adapt the way it cares and supports its service users, at the same time as it has lost large portions of its volunteer workforce and charitable funding streams. The palliative and supportive care usually provided by hospices is premised upon interpersonal relations that emphasise holistic approaches to the quality of life ( Clark, 2014; Clark & Seymour, 1999). The pandemic has provided an almost existential threat to this way of providing care and support as it, initially at least, reduced what was possible and quite literarily shifted the organisational and interpersonal boundaries of palliative care ( Driessen *et al*., 2021).

The stakeholder event allowed us to gain insight into how many of the issues discussed have been experienced within the West Midlands’ hospice community, which is the geographical location for the main study. To balance this regional emphasis, we drew on the expertise of the knowledge exchange panel participants, who came from across the country. The knowledge exchange panel participants also allowed us to contextualise insights and interpretations that were specific to hospice care, and to those that resonated more widely – be that in with the overlaps with primary care in the community or how the pandemic has affected staff across healthcare services.

This knowledge synthesis also provides some insights into the emerging longer-term changes that are taking place in hospice care provision, from the experiments in digital and remote service provision to the acceleration of hospice-at-home initiatives. The expected growth in need for palliative care ( Etkind *et al*., 2017) had already initiated a number of service changes and initiatives, many of which have been accelerated. While the practical necessity of rapid implementation is not in question, what has been gained and lost by circumventing ‘usual’ processes is still to be ascertained, both for individual initiatives and for the wider field of community palliative care.

### Limitations of this knowledge synthesis

The literature discussed here was not collated following a systematic review protocol. It does not include methodological quality checks of the studies undertaken and may be missing relevant studies or reports. But the collaborative review fulfils the aims we set ourselves, which was twofold: first to orientate and sensitise the researchers to the issues they may come across during the main study. Secondly, we aimed to provide all those interested in the impact of the COVID-19 pandemic upon hospices and their service users with an overview of what has and is happening in the hospice sector in the UK.

We should also note that while the list of themes provided here is diverse, we do not suggest it is definitive. What we have provided are a series of interpretive themes that have helped us make sense of the evidence we have considered, so far, in relation to the main study we are conducting. In particular, we hope to have identified particular issues that will allow us to interrogate the systems, processes and experiences of the uneven effects of the pandemic upon those with life-limiting conditions, both as a ‘vulnerable cohort’ and by exploring the different experiences of socio-economic and demographic groups within that population.

Finally, we recognise that our attempts to collaborate with a range of stakeholders was limited by conditions of the pandemic itself. That is, we were not able to get timely or safe access to people with life-limiting conditions or their carers for their input. Nonetheless, our patient and public involvement representatives were able to provide much needed service user perspectives. Not only have their contributions and questions shaped the focus of the main study, but they will be equally involved in the analysis of the in-depth interviews with all four cohorts of the main study: patients, carers, staff and service providers.

## Conclusion

It is evident that hospice care and support services were overlooked at key moments during the pandemic and in policy planning. Hospice services rapidly adapted their ways of working, either bringing new initiatives into place or enacting long held plans. The impact of these changes on hospices has not been fully assessed, but more importantly, it is not known what the effects upon the quality of care and support are for those with life-limiting conditions and those that care for them. That is, the pandemic has presented a new normative and service context in which quality of care and life itself was valued that is, as of yet, poorly understood.

## Data availability

### Underlying data

Transcripts of the knowledge exchange panel contain personally identifiable data. We have permission to store the data at the lead author’s institution. It can be made available upon a reasonable request to the lead author (JM).

### Extended data

Zenodo: ICOH Pre-study stimulus materials, https://doi.org/10.5281/zenodo.5495605

( MacArtney *et al.,* 2021)

This project contains the following extended data:

- Stakeholder event
  ○ Stimulus questions

- Knowledge exchange panel
  ○ Presentation slides from the meeting
  ○ Stimulus materials – potential themes identified form the literature and stakeholder meeting.

Data are available under the terms of the Creative Commons Attribution 4.0 International license (CC-BY 4.0).
