# Supplementary material for: What do we know about the impact of the Covid-19 pandemic on hospices? A collaborative multi-stakeholder knowledge synthesis
Source: AMRC Open Res. 2021 Oct 6;3:23. [Version 1] doi: 10.12688/amrcopenres.13023.1 (PMC11064980; doi:10.12688/amrcopenres.13023.1)
Supplement: Peer review history

# Reviewer report: Reviewer response for version 1

**DOI:** 10.21956/amrcopenres.14098.r26823

**Published:** 2021-11-02

**Authors:** Renske Visser

**Affiliations:** 1 School of Health Sciences, University of Surrey, Guildford, UK

I agree with the other two reviewers that this is a very comprehensive article on the impact of the pandemic on hospices. This could indeed be a baseline paper for others to build on in the future.

 I only have a few minor comments; in the methods section it is stated that Stakeholder engagement focus group was not transcribed. They state: "Verbal consent was provided by the participants for us to record the event on Microsoft (MS) Teams, but not transcribed, so it could be reviewed to ensure no issues, themes or nuances were missed" - I am not sure if I fully understand this, or whether this is a typo and it was in fact transcribed. If the authors could clarify that would be great.

 Other than that I have no major issues with this paper and I strongly encourage it to pass peer review.

Is the work clearly and accurately presented and does it cite the current literature?

Yes

If applicable, is the statistical analysis and its interpretation appropriate?

Not applicable

Are all the source data underlying the results available to ensure full reproducibility?

No

Is the study design appropriate and is the work technically sound?

Yes

Are the conclusions drawn adequately supported by the results?

Yes

Are sufficient details of methods and analysis provided to allow replication by others?

Yes

Reviewer Expertise:

Death Studies

I confirm that I have read this submission and believe that I have an appropriate level of expertise to confirm that it is of an acceptable scientific standard.

# Reviewer report: Reviewer response for version 1

**DOI:** 10.21956/amrcopenres.14098.r26824

**Published:** 2021-10-25

**Authors:** Katherine Kenny

**Affiliations:** 1 Sydney Centre for Healthy Societies, School of Social and Political Sciences, The University of Sydney, Sydney, NSW, Australia

In this article, MacArtney et al. report on the findings of a 'pre-study phase' of a larger ESRC-funded program of research into the experiences, and impact, of COVID-19 vis-a-vis hospice care in the UK. This was carried out in lieu of a systematic review of the literature before embarking on the main study given the rapidly evolving terrain of the COVID-19 response. The article reports on data gained from a three-pronged methodological approach: a review of the emerging literature, a focus group with hospice stakeholders and a collaborative knowledge exchange panel. This approach yielded a range of important, if not entirely unexpected, findings that nevertheless helps build a vital evidence base of how hospices have been affected by the pandemic. Hospice services have often been overlooked or overshadowed in the pandemic response in comparison to the impacts and needs of hospitals and intensive care settings. Yet hospices remain a vital healthcare setting that has experienced a wide range of negative impacts over the last two years. The findings are divided into two broad sections: the first documenting the various ways in which hospices have been impacted by COVID-19, and the second detailing how quality of care has affected, and the changes care delivery has undergone in order to minimize any negative impact. Each of these gives valuable insight into the multifarious ways in which the pandemic has changed what is possible, irrespective of what is needed, and how hospices have adapted at short notice given the resources and constraints at hand. It will have laid important ground for the larger study, and I will be particularly interested in the findings of the in depth qualitative interviews that are to come.

 I was particularly impressed by the innovative methods used to ascertain these findings with very quick turnaround. The multi-stakeholder focus groups and stakeholder panel seem like great ways to engage with service providers and to co-design research. This is a valuable contribution that will be of interest to AMRC Open Research Readers. I have two small reservations that the authors may or may not want to action. The first is that I thought more could be said about how the PPI representatives may and may not speak for the full range of experiences of patients and publics for whom experiences of hospice care during the pandemic no doubt varied greatly. This is mostly a quibble but I thought it could bear a bit more attention. Second, I found the very last sentence of the abstract hard to follow. It recurs as the concluding sentence to the article, where I understood it better. But when I first read it in the abstract, I found it confusing. Again, a very small edit, if the authors are inclined to make it. Otherwise, I fully endorse the findings of this submission.

Is the work clearly and accurately presented and does it cite the current literature?

Yes

If applicable, is the statistical analysis and its interpretation appropriate?

Not applicable

Are all the source data underlying the results available to ensure full reproducibility?

No source data required

Is the study design appropriate and is the work technically sound?

Yes

Are the conclusions drawn adequately supported by the results?

Yes

Are sufficient details of methods and analysis provided to allow replication by others?

Yes

Reviewer Expertise:

Sociology, sociology of health and illness, end of life, qualitative research

I confirm that I have read this submission and believe that I have an appropriate level of expertise to confirm that it is of an acceptable scientific standard.

# Reviewer report: Reviewer response for version 1

**DOI:** 10.21956/amrcopenres.14098.r26821

**Published:** 2021-10-20

**Authors:** Andreas Walker

**Affiliations:** 1 ADG Scientific - Center for Research and Cooperation, Montabaur, Germany

We still know far too little about how the current Covid-19 pandemic will affect the health sector in the medium and long term. If hospitals were and are the focus of the pandemic, we actually know little to nothing about how hospices have experienced the pandemic. It is precisely this knowledge gap that MacArney et al. are trying to fill. Their article "What do we know about the impact of the Covid-19 pandemic on hospices? A collaborative multi-stakeholder knowledge synthesis" is a first approach and synopsis of the topic. Methodologically, the authors approach the issue from different angles: through a review of available literature, which is not yet too extensive, by means of a focus group of hospice stakeholders (including hospice clinicians) and through a collaborative knowledge exchange panel, which identified the topics and issues that provide the most insight into the impact of COVID-19 upon hospice services. The article by MacArtney et al. is therefore first of all a baseline study of the issues and problems that hospices have had to deal with during the pandemic. It starts with the fact that hospices also suffered from a shortage of PPE, medicines and staff. In addition, hospices could no longer fulfil their holistic mission as intended due to visit arrangements for relatives or loss of volunteer support. Hospices also suffered from financial constraints as charity shops had to close or fundraising had to be paused. Communication with patients hampered by the PPE was also challenging for staff. Even though visiting rules in hospices were not as strict as in hospitals, it was an extra communication effort to keep patients' families and carers informed. In addition, however, the article points out that palliative care needs to be further embedded in communities, as palliative care will move from institutions to private homes due to the constraints of the pandemic. There is also a need for different digital literacy, as more remote communication methods would be in use. MacArtney et al. have addressed an important area and highlighted the difficulties of hospice care during the pandemic. It is somewhat unfortunate that the authors did not also point out the advantages and ambivalences associated with the pandemic (less contact with relatives can also mean less stress for the caregiver). Overall, passing peer review is highly recommended.

Is the work clearly and accurately presented and does it cite the current literature?

Yes

If applicable, is the statistical analysis and its interpretation appropriate?

Not applicable

Are all the source data underlying the results available to ensure full reproducibility?

No source data required

Is the study design appropriate and is the work technically sound?

Yes

Are the conclusions drawn adequately supported by the results?

Yes

Are sufficient details of methods and analysis provided to allow replication by others?

Yes

Reviewer Expertise:

hospice care, palliative care

I confirm that I have read this submission and believe that I have an appropriate level of expertise to confirm that it is of an acceptable scientific standard.
